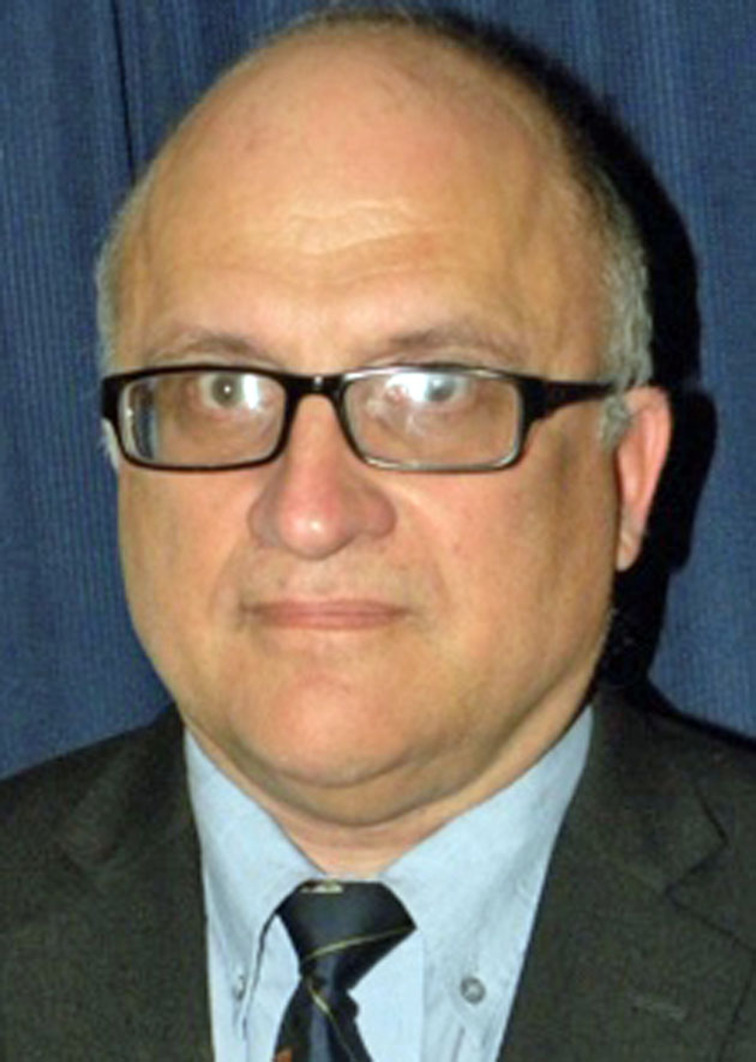# Terephthalic Acid Copolyesters Containing Tetramethylcyclobutanediol for High‐Performance Plastics

**DOI:** 10.1002/open.202100171

**Published:** 2021-08-17

**Authors:** Samarthya Bhagia, Kamlesh Bornani, Soydan Ozcan, Arthur J. Ragauskas

**Affiliations:** ^1^ Biosciences Division Oak Ridge National Laboratory Oak Ridge Tennessee 37831 USA; ^2^ Department of Mechanical Engineering University of Vermont Burlington Vermont 05405 USA; ^3^ Manufacturing Science Division Oak Ridge National Laboratory Oak Ridge Tennessee37831 USA; ^4^ Department of Chemical and Biomolecular Engineering University of Tennessee Knoxville Tennessee 37996 USA; ^5^ Joint Institute of Biological Sciences Biosciences Division Oak Ridge National Laboratory Oak Ridge Tennessee 37831 USA; ^6^ Center for Renewable Carbon Department of Forestry Wildlife and Fisheries University of Tennessee Institute of Agriculture Knoxville Tennessee 37996 USA

**Keywords:** polyester, copolyesters, plastic recycling, terephthalic acid, tetramethylcyclobutanediol, ethylene glycol, cyclohexanedimethanol

## Abstract

There is a need for high‐performance applications for terephthalic acid (TPA) polyesters with high heat resistance, impact toughness, and optical clarity. Bisphenol A (BPA) based polycarbonates and polyarylates have such properties, but BPA is an endocrine disruptor. Therefore, new TPA polyesters that are less hazardous to health and the environment are becoming popular. Tetramethylcyclobutanediol (TMCD) is a difunctional monomer that can be polymerized with TPA and other diols to yield copolyesters with superior properties to conventional TPA polyesters. It has a cyclobutyl ring that makes it more rigid than cyclohexanedimethanol (CHDM) and EG. Thus, TMCD containing TPA copolyesters can have high heat resistance and impact strength. TPA can be made from abundantly available upcycled polyethylene terephthalate (PET). Therefore, this review discusses the synthesis of monomers and copolyesters, the impact of diol composition on material properties, molecular weight, effects of photodegradation, health safety, and substitution of cyclobutane diols for future polyesters.

## Graphical abstract

**High‐performance plastics**: Tetramethylcyclobutanediol containing terephthalic acid based copolyesters can give high heat resistance and impact toughness which are useful in the manufacturing of high‐performance plastic parts.

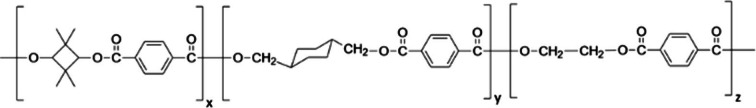

## 1 Introduction

Aromatic diacids and diols can be esterified to make polyesters, and some compositions can yield high mechanical strength and thermal stability, which are useful for making high‐performance components. Terephthalic acid (TPA) is a common diacid monomer used commercially in large quantities to make thermoplastics. The diacid groups of TPA are esterified with aliphatic diols like ethylene glycol (EG), cyclohexanedimethanol (CHDM), and 1,4‐butanediol (BDO) to form polyesters. The homopolymer of TPA with EG is polyethylene terephthalate (PET), and that with BDO is polybutylene terephthalate (PBT). PET can contain minor quantities of cyclohexanedimethanol (CHDM) (co‐monomer) to lower the crystallinity for easier thermoforming and to impart clarity to PET for making plastic bottles.^[1]^ Glycol‐modified PET (PETG) contains both EG and significant quantities of CHDM, but CHDM content is <50 mol % of the total diol component. PET and PETG have glass transition temperatures (*T_g_*) near ∼80 °C and notched Izod impact strength around 35–80 J/m. TPA‐CHDM homopolyester is known as PCT, which has *T_g_* ∼88 °C and high impact strength (∼1200 J/m) but is difficult to thermoform into articles.^[2]^ Therefore, EG (<50 mol % of total diol) is added to TPA‐CHDM to make glycol‐modified PCT (PCTG) copolyester which is easier to thermoform. A portion of TPA can be replaced by its isomer, isophthalic acid (IPA), to get a PCTA copolyester.^[3]^ When TPA is esterified with an aromatic diol like bisphenol A (BPA), the resulting polyester (polyarylate) can have very high *T_g_* (180–210 °C), melting temperature (*T_m_* 350 °C),^[4]^ and good impact strength (224 J/m)^[5]^ due to the benzene rings that provide rigidity to the structure in comparison to aliphatic diols. The thermal stability is even higher than BPA polycarbonate (PC), which has *T_g_* ∼145–150 °C and but PC has a very high notched Izod impact strength of ∼930 J/m.^[6]^

**Figure 1:**
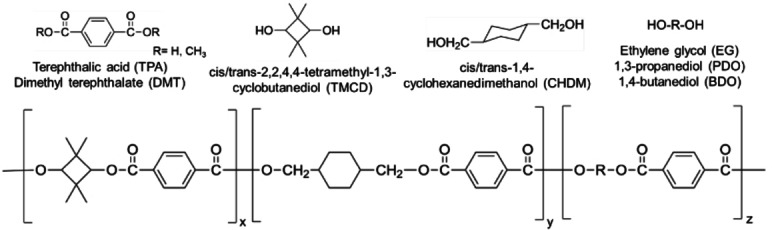
Copolyester made from aromatic diacids like terephthalic acid (TPA) and diols like tetramethylcyclobutanediol (TMCD), ethylene glycol (EG), and cyclohexanedimethanol (CHDM).

Bisphenol A (BPA) is a known endocrine disruptive chemical (EDC). Therefore, BPA‐free polyesters are gaining momentum as replacements in the market place, especially in biomedical applications and food contact materials.^[7]^ BPA alternatives include cyclobutanediol (CBDO) derivatives like *cis/trans*‐2,2,4,4‐tetramethyl‐1,3‐cyclobutanediol (TMCD) that maintain structural rigidity without loss in other properties. Replacement of the aromatic diol with a rigid cycloaliphatic diol increases photostability and solvent resistance.^[8]^ A recent study shows that 4,4′‐bibenzoate‐CHDM polyester can achieve *T_g_* of ∼135 °C, which gets close to BPA polycarbonate (PC) commodity plastic that has *T_g_* of ∼145 °C.^[9]^ Monomers like TPA, TMCD, CHDM, and EG can be polymerized to make segmented copolyesters that have good *T_g_*, impact strength and clarity (Figure 1). Eastman Chemical Company synthesizes such copolyesters under their Tritan® brand. The abundance of PET waste presents an opportunity for upcycling it by solvolysis to recover TPA, EG and CHDM monomers for synthesizing high‐performance copolyesters instead of neosythesis of monomers from petroleum. Such copolyesters that have been made by upcycling or recycling and serve as BPA substitutes are gaining demand.

Therefore, this review presents synthesis and properties of TMCD containing TPA‐based copolyesters that can be made to yield high *T_g_* (>100 °C), impact strength (600–1100 J/m), flexural modulus (2–2.5 GPa), Rockwell hardness (80–100), solvent resistance and optical clarity.^[6]^ The impact of monomer ratios on properties of the polyesters, molecular weight analysis, effect of aging, and applications are discussed. The latter sections review recent health and environmental safety findings based on endocrine biological assays and TMCD alternatives for making new copolyesters of this class.

## 2 Production of Monomers

### 2.1 Terephthalic Acid and Ethylene Glycol from Lysis of PET

The neosynthesis of TPA and its methyl ester (DMT) can be accomplished by the oxidation of petroleum‐derived *p*‐xylene (Amoco process) or indirectly via methyl toluate over Co(II) or Mn(II) catalysts.^[10]^ The ester form makes it easy to distill and recover monomers in highly pure form, which is essential for subsequent high molecular weight polycondensation reactions.^[10]^ Instead, recycling of PET can be done to recover TPA and EG by chemical or biological methods due to commercial abundance. Ester linkages can be cleaved by neutral, acidic, alkaline hydrolysis and alcoholysis. Hydrolysis with steam above 245 °C naturally lowers the pH to 3.5–4 from TPA formation, and the reaction rates can be increased by adding acetates of Zn, Ca, or Mn. Acidolysis uses 67–87 % H_2_SO_4_ to cleave TPA and EG, followed by neutralization and purification. Alkaline hydrolysis is carried out using 4–20 % NaOH or aqueous NH_3_ that form salts of TPA. Methanolysis at 180–280 °C at 2–4 MPa using transesterification catalysts like Zn(CH_3_COO)_2_ converts PET into dimethyl terephthalate (DMT), EG, and minor quantities of side products like methyl(hydroxyethyl) terephthalate (MHET). Glycolysis of PET using ethylene glycol at 180–250 °C and 0.1–0.6 MPa for 0.5–8 h with Zn(CH_3_COO)_2_ produces bis(hydroxylethyl)terephthalate (BHET).^[1]^

Esterases produced by bacteria and fungi can degrade polyesters, but the process is slower than chemical catalysis. Bacteria such as *Ideonella sakaiensis*, *Thermobifida fusca*, *Thermobifida cellulosilytica*, *Thermobifida alba*, *Bacillus subtilis*, *Thermomonospora curvata*, *Saccharomonospora viridis* and fungi *Fusarium solani*, *Humicola insolens*, and *Aspergillus oryzae* are known to carry PET hydrolytic enzyme (PHE).^[11]^ *Ideonella sakaiensis* can grow on PET as the sole carbon source and produces CO_2_ and water. Its esterases can be isolated for the recovery of TPA and EG from PET.^[11]^ However, enzyme accessibility is limited due to *T_g_* of PET (∼70 °C), and heat‐stable enzymes are needed for efficient enzymatic hydrolysis. Thermal inactivation *of T. fusca* and *T. alba* PETases was reduced by adding Ca^2+^ ions or replacing the calcium‐binding site with disulfide bridges.^[12]^ Recently, *Ideonella sakaiensis* 201‐F6 PETase gene was expressed in marine algae *Phaeodactylum tricornutum* host for PET degradation in saltwater at near room temperature.^[13]^

### 2.2 1,4‐Cyclohexanedimethanol (CHDM)

**Figure 2:**
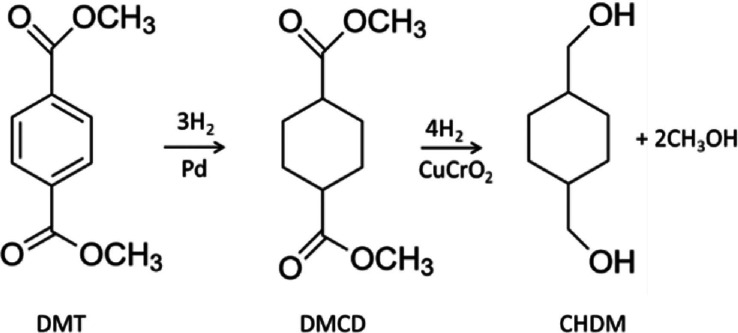
Synthesis of CHDM.^[14]^

CHDM can be produced from DMT by hydrogenation in two steps: hydrogenation of DMT over palladium to get dimethyl hexahydroterephthalate (DMHT) (dimethyl 1,4‐cyclohexanedicarboxylate (DMCD)) at 30–48 MPa and 160–180 °C and then hydrogenation of DMHT over copper chromite^[14]^ (Figure 2). Approximately 99 % pure CHDM is recovered after distillation of methanol and low‐boiling compounds. The process produces an isomer ratio of 30/70 *cis/trans* CHDM.

### 2.3 Substituted Cyclobutanediols (CBDO) and Tetramethylcyclobutanediol (TMCD)

**Figure 3:**
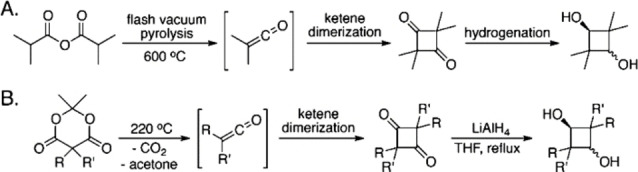
(A) Synthetic procedure used commercially for the preparation of 2,2,4,4‐tetramethyl‐1,3‐cyclobutanediol (TMCD); (B) General route to make cyclobutanediols from Meldrum's acid derivatives. Reprinted (adapted) with permission from Burke et al.^[16]^ Copyright 2012 American Chemical Society.

Synthesis of tetramethyl substituted CBDO (TMCD) was first reported by Staundinger,^[15]^ who observed the spontaneous dimerization of dimethylketene. Despite the versatile properties of a monomer, producing TMCD and other substituted CBDOs in large quantities is a complex task due to its novel chemistry, in part. Synthesis of TMCD is carried out commercially by vacuum flash pyrolysis of isobutyric anhydride to form dimethyl ketene, dimerization of ketene to tetramethylcyclobutanedione, and then hydrogenation to form the diol^[16]^ (Figure 3). The use of ruthenium catalyst for the hydrogenation step has been shown to obtain control over the isomers produced, with a 50/50 *cis/trans* ratio.^[17]^ The *trans* isomer has a lower melting temperature (148 °C) than *the cis* isomer (160–163 °C) due to its planar conformation, which enables its purification via acid dehydration.[^18^, ^19^, ^20^] Although the synthetic route is effective, it limits the synthesis of diverse substituents on CBDO due to the lack of availability of substituted anhydrides.^[16]^ Therefore, another route has been developed based on substituted 5,5′‐dialkyl acid derivatives, known as Meldrum′s acid. These dialkyl acid derivatives facilitate the formation of cyclic compounds through Diels‐Alder cycloaddition followed by reduction to get substituted CBDO products. These reactions can be carried out at lower temperatures than flash pyrolysis, and acetone and CO_2_ side products can be removed easily.^[16]^

## 3 Synthesis of the Polyester

### 3.1 Polymerization Conditions

**Figure 4:**
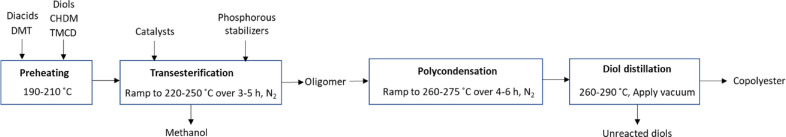
Process of polymerization.

The equimolar ratio of total diacid and total diol react to form the final polyester. Diols are added in excess (1.5 to 2 times higher) to assure complete esterification of the diacid groups with diols. The monomers are first converted into oligomers (transesterification stage) and then into high molecular weight polyesters (polycondensation stage). The unreacted diols are distilled to complete the polymerization (Figure 4). 80–100 mol % of total acid TPA and isomer 0–20 mol % isophthalic acid (IPA) and diols like TMCD, CHDM, and EG are usually the main monomers. Other modifying monomers are listed in Table 1.

**Table 1:** Dicarboxylic acid and diol monomers for synthesis of TPA‐based copolyesters.[^21^, ^22^]

Diacids or their alkyl esters	Diols
Terephthalic acid, or in ester form (Dimethyl, dipropyl, diisopropyl, dibutyl, diphenyl‐) (TPA, DMT)	cis/trans‐ 1,4‐Cyclohexanedimethanol (CHDM)
	cis/trans‐2,2,4,4‐Tetramethyl‐1,3‐cyclobutanediol (TMCD)
*Modifying minor diacids (<20 mol % of total diacid)*	Ethylene Glycol (EG)
Isophthalic acid (IPA)	
4,4’‐biphenyldicarboxylic acid	*Modifying minor diols (<5 mol % of total diol)*
1,4‐, 1,5‐, 2,6‐, 2,7‐, naphthalenedicarboxylic acid (NDA)	1,2, 1,3‐, ‐propanediol
Trans‐4,4’‐stilbenedicarboxylic acid	1,4‐butanediol
Malonic acid	Neopentyl glycol
Succinic acid	1,5‐pentanediol
Glutaric acid	1,6‐hexanediol
Adipic acid	P‐xylene glycol
Pimelic acid	Polyethylene glycols
Suberic acid	Polytetramethylene glycols
Azelaic acid	
Dodecanedioic dicarboxylic acids	
Indan‐1,3‐ and phenylindan dicarboxylic acids

The reactions are carried out in an inert atmosphere like nitrogen or argon to prevent oxidation. Transesterification is carried out at a lower temperature than polycondensation. DMT is first melted at 190–210 °C in the reactor. Then the temperature is raised to 220–250 °C for 1–4 h and 45–550 kPa to carry out transesterification of DMT with diols like TMCD and CHDM, which produces an oligomer and methanol. Removal of methanol by distillation drives oligomer formation. The oligomer is then further polymerized with the diols at 260–275 °C for 4–6 h to produce the high‐molecular weight copolyester. Finally, the temperature is raised to 275–290 °C, and a vacuum is applied near the end of the reaction to distill out the unreacted diols (Figure 4). Helicone‐type impeller designs can be used for such high melt viscosity polymerizations.^[6]^ TPA‐TMCD/CHDM polymerization can form poly(1,4‐cyclohexylene dimethylene terephthalate) (PCT) as a side product whose precipitation can terminate the polymerization. Precipitation can be avoided by keeping reactor temperature close to T_m_ of PCT (∼290 °C), but temperatures >270 °C accelerate the degradation of monomers and cause yellowing. Therefore, a better strategy involves sequential addition of TMCD at >50 mole% of the diol component and then CHDM as this reduces precipitation of PCT. At a large scale, excess of TMCD with diacid with a diol:diacid ratio>1 (like 1.2) is added to the first stage, and then remaining CHDM with diacid with diol:diacid ratio <1 is added in the next stage.^[23]^ Reaction progress is monitored by measurements of intrinsic viscosity, which is correlated to molecular weight.^[21]^ Usually, a 50/50 ratio of cis/trans TMCD is reported. The cis/trans ratio of CHDM can be varied from 25/75 to 35/65.^[22]^

### 3.2 Catalysts and Phosphorous Compounds

Tin and manganese‐based catalysts are effective at polymerizing high concentrations of TMCD in TPA‐TMCD/EG systems, but titanium‐based catalysts alone cannot do the same due to lower reactivity with TMCD. However, tin catalysts impart a yellow color to the polymer. Therefore, a combination of Sn/Mn with Ti and phosphorous compounds can be used. It is hypothesized that Ti can coordinate with cisTMCD isomer which reduces its catalytic activity.^[24]^ Tin compounds include dialkyl tin dihalides, diaryl tin oxides, tin alkoxides, and those with C−Sn linkages like dialkyl tin and dialkyl tin oxides. Cobalt, antimony, germanium, lithium, and aluminum catalysts can also be used in combination.[^21^, ^22^] The concentrations of these compounds in polymerization range in 10–50 ppm Ti atoms, 10–100 ppm Sn or Mn atoms, and 100–200 ppm phosphorous compound based on polymer weight.^[25]^ The rates of reaction of TMCD with TPA at 240 °C decreased in the order: SnOBu_2_>Co(OAc)_2_>Mn(OAc)_2_>Zn(Ac)_2_.^[24]^

Alkyl and aryl phosphorous compounds like triphenyl phosphate, bis(2,4‐dicumylphenyl)pentaerythritol diphosphite, potassium, and zinc phosphates act as thermal or color stabilizers to obtain a colorless polymer. In TPA‐(30–40 %TMCD)/(30–70 % EG) system polycondensation occurs at 265–275 °C for 160–230 min with Mn(OAc)_2_ and Ti (IV) isopropoxide catalysts and Merpol A phosphorous compound (CAS#37208‐27‐8), the polyester had L* of 90 to 94, a* of −0.36 to −0.94 and b* of 3.64 to 7.19 in CIELAB color space, where L* (lightness coordinate): 0 is black, 100 is white, a* (green/red): <0 is green, >0 is red and b* (blue/yellow): <0 is blue, >0 is yellow. When TMCD is >25 %, a combination of Sn and P compounds alone cannot be used as the b* values are 10–20. Ti and P compounds give b* values of 4–5, and Sn, Ti, and P give b* values of 5–9.^[21]^ Phosphorous stabilizers also reduce foaming, off‐gassing and help in building intrinsic viscosity.^[2]^ In the absence of phosphorous compounds, 20 % PDO or BDO gave lower yellow‐colored TPA‐80 %TMCD/20 %diol copolyester than 20 % EG when dibutyl tin oxide and titanium tetrabutoxide catalysts and Irganox 1098 free radical inhibitor were used.^[26]^

## 4 Impact of Monomer Composition on Properties of the Copolyesters

Appropriate ratios of diacid and diol monomers are important for achieving good thermal stability and mechanical strength while assuring that the polymer has suitable viscosity, clarity and can be thermoformed without degradation. Various modifying diacid and diols can be added in addition to the primary monomers to tailor the physical properties as required by the application (Table 1). Diacid monomers that contain a benzene ring like terephthalic acid provide structural rigidity and high glass transition temperature. Minor quantities of aromatic diacids like isophthalic acid (IPA), naphthalenedicarboxylic acid (NDA), or stilbene dicarboxylic acid can be added along with TPA at <20 mol % of total diacid moles. Greater than 80 mol % of total diacid of TPA is usually needed to maintain high impact strength and optimum melt viscosity for injection molding and extrusion. Aliphatic diacids of 2–16 carbon atoms like malonic acid, succinic acid, glutaric acid, adipic acid have to be kept below 10 mol % as they lower *T_g_* and HDT.^[27]^ Diols can be a combination of TMCD+CHDM or TMCD+CHDM+another aliphatic diol like EG, 1,3‐propanediol (PDO), 1,4‐butanediol (BDO) or neopentyl glycol.^[28]^

Simulations found that the root mean square end to end distance (47885 and 42226 Å^2^), characteristic ratio (41.8 and 36.9), and persistence length (40.8 and 29.6 Å) of TPA‐*trans*TMCD were much higher than TPA‐*cis*CHDM (11083 and 11495 Å^2^), (7.4 and 7.7) and (6.2 and 6.4 Å) at 300 and 448 K. This increase in chain dimensions by TMCD give the TPA‐*trans*TMCD copolyester chain rigidity and stiffness in comparison to TPA‐*cis*CHDM copolyester chain. Due to steric hindrance between the methyl groups of TMCD and carbonyl oxygen, the TPA‐TMCD unit′s torsional flexibility is limited to 5 and 95°. However, it was also found that TPA‐*50/50cis/trans*TMCD chain had similar dimensions as TPA‐*30/70cis*/*trans*CHDM because of the higher *cis* content of the TPA‐TMCD chain. These *cis/trans* ratios of TMCD and CHDM are typical of those resulting from chemical synthesis.[^29^, ^30^]

CHDM increases the mobility of polymer chains as the flipping of cyclohexane confirmation causes relaxation, which increases ductile behavior. Aliphatic diols like EG, PDO, and BDO are even more flexible than CHDM due to the absence of ring structure.^[30]^ *cis/trans* ratio of CHDM affects *T_m_* as the softening point of *cis*CHDM is 43 °C, and *trans*CHDM is 67 °C. TPA‐*30/70cis*/*trans*CHDM polymer (PCT) and TPA‐EG polymer (PET) have T_g_ of 88 and 80 °C and T_m_ of 300 and 260 °C, respectively.^[31]^ Table 2 shows the effect of the major diol and minor diol composition on thermal and mechanical properties.

**Table 2:** Effect of Diol Composition on Thermal and Mechanical Properties of TPA Homo/Copolyesters.[^3^, ^6^, ^21^, ^22^, ^23^, ^24^, ^25^, ^26^, ^32^]

Major Diol [mol %]	Minor Diol [mol %]	Thermal Properties [°C]	Mechanical Properties
100 EG (PET)	N/A	T_g_ 78–80, HDT 61, T_m_ 260	Impact 35.2 J/m, Hardness 76
96.5 EG	3.5 TMCD	T_m_ 254	–
95 EG	5 TMCD	T_m_ 245	–
90 EG	10 TMCD	T_m_ 224	–
87 EG	13 TMCD	T_g_ 89, HDT 66	Impact 41.1 J/m, Hardness 76
77 EG	23 TMCD	T_g_ 93.1, HDT 70	Flex. mod. 2.360 GPa, Impact 55 J/m, Yield strength 55 MPa, Break stress 52 MPa, 4 % yield strain, 333 % break strain, Young′s mod. 2.36 GPa
75 EG	25 TMCD	T_g_ 95.6	–
69 EG (PETG)	31 CHDM	T_g_ 80, HDT 64, T_m_ 265	Impact 83 J/m
66 EG	34 TMCD	T_g_ 101, HDT 80	Impact 83.8 J/m, Hardness 80
65 EG	35 TMCD	T_g_ 105, HDT 82	–
64 EG	36 TMCD	T_g_ 106.5	–
58‐68 EG	32–42 TMCD	T_g_ 100–110	Impact: 30–80 J/m, flexural mod. 2 GPa
100 CHDM (PCT)	N/A	T_g_ 88, HDT (at 264 psi) 60, T_m_ 290–300	Impact 1222.9 J/m, Hardness 71
60–80 CHDM	20–40 TMCD	T_g_ 100–130	–
78 CHDM	22 TMCD	T_g_ 106	–
73 CHDM	27 TMCD	T_g_ 113	Impact 877 J/m
69 CHDM	31 TMCD	T_g_ 116	Impact 807 J/m
62 CHDM (PCTG)	38 EG	T_g_ 86	–
57.2 CHDM	42.8 TMCD	T_g_ 133	–
58 CHDM	42 EG	HDT 67	Impact 1532.6 J/m, Hardness 60
56 CHDM	44 TMCD	T_g_ 128	
CHDM	TMCD (commercial)	T_g_ 107.3	44.2 MPa yield strength, 59.0 MPa break strength, 5.8 % yield strain, 188.4 % break strain, Young′s modulus 1.488 GPa, Impact 1099 J/m
100 TMCD	N/A	T_g_ 174, T_m_ 317–325	–
87 TMCD	13 PDO	T_g_ 168	–
84 TMCD	16 EG	HDT 118	Impact 137.8 J/m, Hardness 103
78 TMCD	22 EG	T_g_ 155	
78 TMCD	22 PDO	T_g_ 150, HDT (at 264 psi) 114	Impact 390 J/m, Young′s mod.1.83 GPa, yield strength 45.5 MPa
78 TMCD	22 BDO	Tg 145, HDT (at 264 psi) 105	Impact 280 J/m, Young′s mod. 1.84 GPa, yield strength 31 MPa
72 TMCD	28 PDO	T_g_ 136	–
72 TMCD	28 BDO	T_g_ 129	–
69 TMCD	31 EG	HDT 108	Impact 611.4 J/m, Hardness 95
65 TMCD	35 PDO	T_g_ 122	–
64 TMCD	36 EG	T_g_ 141, HDT 102	Impact 662.2 J/m, Hardness 94
64 TMCD	36 BDO	T_g_ 119	–
57 TMCD	43 PDO	T_g_ 112	–
53 TMCD	47 PDO	T_g_ 101	–
50 TMCD	50 EG	HDT 90	Impact 129.8 J/m, Hardness 90
30 TMCD+30 CHDM	40 EG	T_g_ 106–118	–
100 BDO (PBT)	N/A	T_g_ 52, T_m_ 228	–
100 PDO	N/A	T_g_ 59, T_m_ 235	–
60 PDO	40 TMCD	T_g_ 87	–

T_g_: glass transition temperature, T_m_ melting temperature, Impact: Notched Izod impact strength at 23 °C, Hardness: Rockwell L, HDT: Heat deflection temperature at 264 psi (1.82 MPa). TPA: terephthalic acid, EG: ethylene glycol, CHDM, 1,4‐cyclohexanedimethanol, TMCD: 2,2,4,4‐tetramethyl‐1,3‐cyclobutanediol, PDO: 1,3‐propanediol, BDO: 1,4‐butanediol. PET: polyethylene terephthalate, PETG: CHDM‐modified PET, PCT: polycyclohexylenedimethylene terephthalate, PCTG: EG‐modified PCT, PBT: Polybutylene terephthalate

### 4.1 TPA‐EG/TMCD copolyesters (TMCD modified PET)

**Figure 5:**
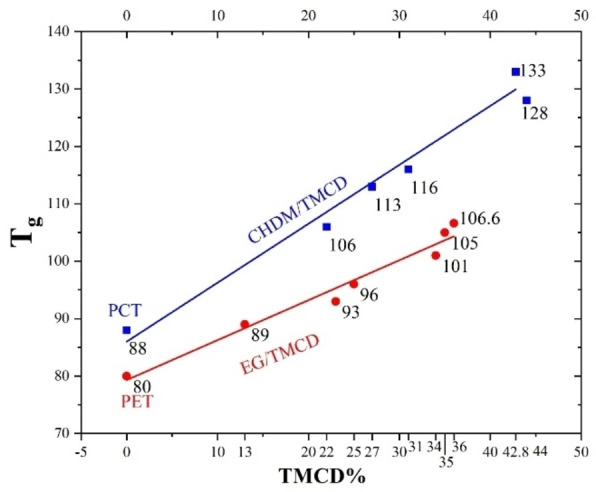
Effect of increasing TMCD content on *T_g_* in PCT and PET type copolyesters (based on Table 2 data)[^6^, ^21^, ^24^, ^25^, ^33^]

Impact strength decreases and hardness increases on increasing the EG content in TPA‐EG/CHDM (PETG) polyester. While the PETG polyester can provide hardness and heat resistance, the impact strength (notched Izod toughness <100 J/m) is lower than that needed from high‐performance plastics.^[3]^ Generally, there is a tradeoff between impact toughness and heat resistance.^[24]^ However, TMCD can increase the impact strength without lowering hardness and heat resistance in the right diol ratio. Increasing TMCD from 23 to 35 mol % of total moles of diol in TPA‐EG/TMCD copolyester increased the *T_g_* from 93 to 105 °C (Figure 5) and HDT from 70 to 82 °C and decreased the intrinsic viscosity at 25 °C from 0.63 to 0.59 dL/g^[25]^ in 40/60 phenol/tetrachloroethane. TPA‐(30–70 %)TMCD/(30–70 %)EG copolyester can have >70 Rockwell L hardness, >70 °C HDT (at 1.82 MPa) and >53.4 J/m notched Izod strength. This polyester gives the best properties when TMCD is 64–69 mol %, and EG is 31–36 mol % that yields a 94–95 Rockwell L hardness, 102–108 °C HDT at 264 1.82 MPa, and 609–667.5 J/m notched Izod strength.^[3]^ High melt strength and low melt viscosity are desirable for making components by blow molding at high rates. Compared to the conventional PETG plastic (TPA‐EG/CHDM), adding just 5 mol % TMCD can substantially increase melt strength and decrease the sagging of parisons during blow molding that gives walls of more even thickness.^[3]^

### 4.2 TPA‐CHDM/TMCD Copolyesters (TMCD Modified PCT)

Homopolymer made only from TPA and CHDM (PCT) has high thermal stability (*T_g_* 88 °C, *T_m_* 300 °C, *T_cc_* 227 °C) and exceptional impact strength (notched Izod>1000 J/m), but it is difficult to mold as polymer starts degrading near its *T_m_* and PCT has a fast crystallization half‐time (<5 min at 170 °C).^[31]^ The addition of TMCD to TPA‐CHDM (PCT) polyester increases *T_g_* (Figure 5), decreases the temperature of cold crystallization (*T_cc_*), decreases melting temperature (*T_m_*), and increases crystallization half time (*t* ^*1/2*^), which makes it easier to thermoform the polyester. By adding *50/50cis/trans*TMCD to PCT (PCTT) and increasing its content from 4.4 to 9.1 mol %, *T_g_* changed from 92 to 98 °C, *T_cc_* from 203 to 176 and *T_m_* from 280 to 270 °C.^[34]^ *t* ^*1/2*^ increased from 15 to 47 min (at 170 °C) and melt viscosity (at 1 rad/s, 290 °C) reduced from 5649 to 1736 poise when *50/50cis/trans*TMCD was increased from 15 to 23 mol % of total diol, respectively.^[6]^ EG does not provide the same effect on *t* ^*1/2*^ as does TMCD. When 20 % of *31/69cis/trans*CHDM was replaced with EG or TMCD in TPA‐CHDM system (PCT), *t* ^*1/2*^ at 170 °C was only 1.4 min with EG but 23.3 min with TMCD (*50/50 cis/trans*). *cis/trans* ratios of TMCD appear to only affect *t* ^*1/2*^ significantly below 160 °C (*98/2cis/trans*TMCD: 11.6–55.0 min vs. *5/95cis/trans*TMCD: 8.1–25.4 min).^[35]^ Moreover, the transition temperature from brittle to ductile failure mode (*T_bd_*) can be lowered by increasing TMCD content in TPA‐CHDM (PCT) based systems. *T_bd_* were 18 and 26 °C on the addition of EG at 38 and 69 mol % of total diol, but they were −5 and −12 °C on addition 22 and 42.8 mol % TMCD, respectively.^[6]^ In the range of 60–80 %CHDM/20‐40 % TMCD, the TPA‐CHDM/TMCD (PCTT) copolyester can achieve high notched Izod impact strength 700–900 J/m and *T_g_* 105–120 °C. These polyesters were amorphous as DSC melting peaks were absent.^[36]^ Since TMCD occupies more space than CHDM and EG in TPA polyesters, TMCD copolyesters have higher oxygen transmission rates (OTR). OTR of TPA‐TMCD/CHDM, TPA‐CHDM polyester (PCT), TPA‐EG/CHDM and TPA‐EG (PET) were 137, 52.6, 25.8 and 14.3 cc‐mil/100 in^2^.day.atm, respectively, in dry conditions.^[37]^

### 4.3 TPA‐TMCD/Linear Diols (EG, PDO, BDO) (Glycol‐Modified TPA‐TMCD)

Homopolyester made only from TPA and TMCD is semi‐crystalline and has a very high T_g_ (174 °C) and T_m_ (>310 °C). TPA‐*cis*TMCD and TPA‐*trans*TMCD have *T_m_* 296–308 °C and >350 °C, respectively.^[6]^ Replacing the portion of TMCD with EG lowers *T_g_*. 20 and 40 mol % EG decreased *T_g_* to 155 and 142 °C. TPA‐TMCD/EG copolyesters were amorphous as DSC did not see a melting peak in 1^st^ or 2^nd^ heating curves at 10 °C/min.^[26]^ TPA‐TMCD/EG copolyesters with>85 mol % TMCD of diol component have high viscosities that make molding difficult. EG range can be 15–75 mol % of total moles of diol. TPA‐TMCD/EG polyesters with <15 mol % of EG have low hardness and heat resistance, but those with >75 mol % EG do not have sufficient impact strength. TPA‐TMCD(42 %)/EG(58 %) copolyesters, where DMT has been obtained from recycled PET, have high thermal stability, stiffness, optical clarity, and durability.^[25]^

**Figure 6:**
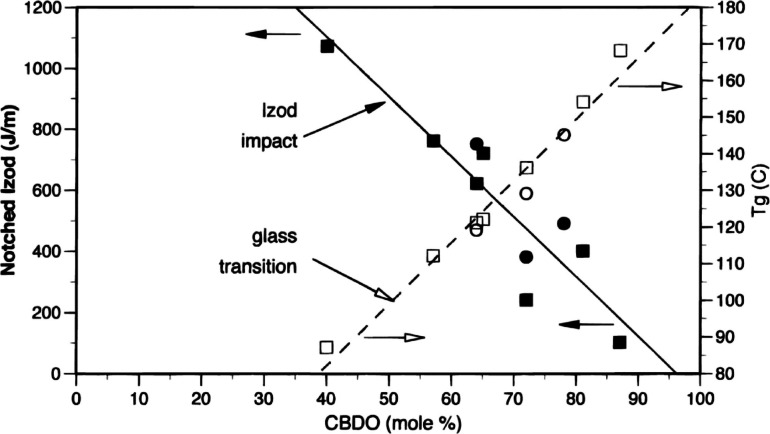
Effect of TMCD (CBDO) content on *T_g_* (open symbols) and notched Izod impact (solid symbols) for TPA copolyesters with 1,3‐propanediol (squares) and 1,4‐butanediol (circles). Reprinted (adapted) with permission from Kelsey et al.^[24]^ Copyright 2000 American Chemical Society.

In TPA‐TMCD/(PDO or BDO) copolyesters, *T_g_* increased, and impact strength decreased on increasing TMCD content from 40 to 90 mol %. Notched Izod impact strength (1070 J/m) was highest at 40 mol % TMCD content^[24]^ (Figure 6). Although TMCD increases the rigidity of the chain, the polyester is not brittle, and polyester containing TMCD in the 50–80 % range can have both high *T_g_* (>100 °C) and toughness (550–800 J/m). Higher *trans* content in TMCD can increase crystallinity as 80 %/20 % TMCD/PDO with *39/61cis/trans*TMCD showed melting transitions and only partial solubility in CH_2_Cl_2_ compared to *48/52cis/trans*TMCD. Lowering the TMCD content to 65 %/35 % TMCD/PDO made the polymers amorphous and soluble in CH_2_Cl_2_.^[24]^

In TPA‐TMCD/PDO polyesters, increasing the content of *cis*TMCD isomer increases optical clarity. Copolyesters that used *trans*‐rich TMCD produced a translucent material compared to highly transparent copolyesters that used *cis*‐rich TMCD. This result was because *cis/trans* TMCD and *cis*‐rich TMCD polyesters were amorphous while *trans*‐rich TMCD polyester was semi‐crystalline and showed peaks at 5.735 and 5.284 Å in XRD. The *T_g_* of copolyesters made from *cis*‐rich, 43/57cis/*trans* mixture and *trans*‐rich TMCD were 99.4, 84.5, and 69.3 °C, the Izod impact strengths were 1090, 944, and 841 J/m, respectively. Models indicate that *cis*TMCD polyester has a coiled structure that helps absorb impact energy like a spring for ballistic armor, while *trans*TMCD polyester is linear, which may be better for yielding fibers of high tensile strength.^[38]^ The same trend between the *cis/trans* ratio of TMCD and *T_g_* has been found in TPA‐TMCD/CHDM systems as well. In TPA‐44 %TMCD/56 %CHDM, using 0.72 *cis/trans* ratio gave *T_g_* 131 °C, while 0.36 *cis/trans* ratio gave *T_g_* 118 °C.^[6]^

## 5 Molecular Weight

GPC and NMR can determine the molecular weight of these copolyesters. Dichloromethane with 10–30 % hexafluoroisopropanol (HFIP) is a good solvent for GPC and intrinsic viscosity measurement. HFIP is an excellent polar protic solvent for the solubilizing of terephthalic acid class of polyesters.^[39]^ The intrinsic viscosity of the copolyesters are in the range of 0.5–0.8 dL/g in phenol/tetrachloroethane at 23 °C.^[23]^ TPA‐TMCD/CHDM copolyester (Tritan TX1000) has weight‐average molecular weight (*M_w_*) of 20 kDa, number‐average (*M_n_*) 10.5 kDa and dispersity (*M_w_/M_n_*) of 1.97.^[32]^ A commercial Tritan (TPA‐TMCD/CHDM) polymer had 53.1 kDa *M_w_* and 26.5 kDa *M_n_* ^[16]^ and a TMCD modified PCT (PCTT) (TPA‐CHDM/TMCD) film had 64.5 kDa *M_w_* and 28.9 kDa *M_n_* from GPC based on polystyrene standards in 70/30 CH_2_Cl_2_/HFIP mobile phase.^[39]^ This result is in the range of other TPA copolyesters like TPA‐62 %CHDM/38 %EG (PCTG), which has *M_w_* 45.7 kDa and *M_n_* 18.7 kDa^[40]^ and PET which has *M_w_* 49.5 kDa and *M_n_* 20.1 kDa^[41]^ by GPC. For TPA‐TMCD/CHDM polymer, the degree of polymerization (DP) can be determined by ^1^H NMR in CHCl_3_‐d/trifluoroacetic acid‐d by dividing the sum of integrals of *cis* (4.75 ppm) and *trans* (4.91 ppm) methine peaks by end group (4.05 ppm) for TMCD units (DP_tmcd_) and the sum of integrals of *cis* (4.43 ppm) and *trans* (4.32 pm) methyl peaks by end group (4.05 ppm) for CHDM units (DP_chdm_). The average number‐average molecular weight (*M_n_*) is M_tmcd_DP_tmcd_+M_chdm_DP_chdm_. Molecular masses M_tmcd_ and M_chdm_ of CHDM and TMCD residues are both 274 g/mol. ^13^C NMR can be employed for calculating *cis*/*trans* ratio (81.9/82.9 ppm for TMCD and 34.3/36.9 ppm for CHDM) and dyad sequences (133.7 from TPA).^[42]^

## 6 Impact of Ageing

TPA‐TMCD/CHDM (Tritan) copolyester was exposed for 2 years behind a glass wall in an enclosure with 0–58 °C temperature variance and 5–27 MJ/m^2^ radiant exposure at 61.4±9.4 % relative humidity. *T_g_* (2^nd^ DSC heating scan) decreased from 92 to 80 °C at the end of the exposure. The tensile yield strength increased from 45 to 51 MPa, possibly due to enthalpic relaxation. The copolyester′s Charpy notched impact strength decreased from 105 to 2 KJ/m^2^ beyond 60 days compared to PET and PETG 3–5 to 1.5 KJ/m^2^. FTIR showed significant photodegradation as the carbonyl peak shifted from 1723 to 1714 cm^−1^ due to lysis of the ester bond.^[43]^ In another study, this copolyester underwent accelerated aging at 40–80 °C for 123 days. Charpy impact strength dropped from 63 to 15 KJ/m^2^ when enthalpic relaxation was maximized to ∼1 J/g (beyond 500 h at 80 °C). However, Young′s modulus increased from 1.47 to 1.59 GPa on aging for 42 days irrespective of temperature (40–80 °C), and tensile strength at yield increased from 46 to 47 MPa, 48 to 52 MPa, and 51 to 58 MPa on aging for 42 days at 40, 60 and 80 °C, respectively.^[42]^ The yellowness index after 2500 h of UV exposure (340 nm) was markedly lower for TPA‐TMCD/BDO copolyester (+29 %) than commercial polycarbonate (+2800 %) in the absence of any UV stabilizer. The notched Izod impact strength of the copolyester decreased from 390 to 110 J/m (−72 %) while that of polycarbonate decreased from 990 to 70 J/m (−93 %) after UV ag.

## 7 Additives for Modifying Properties

Several additives can be added to improve performance, like impact modifiers, UV and thermal stabilizers, hydrophobicity modifiers, surface friction, and slip agents, antimicrobial substances, dyes and pigments, toners, antistatic agents, and flame retardents.^[44]^

### 7.1 Impact Modifiers

Impact strength can be increased by adding elastomers and having one or more polymer segments below RT. Modified polyolefins (Elvaloy, Lotader), thermoplastic elastomers (Kraton, thermoplastic urethane), and core‐shell polymers (cross‐linked acrylates and methacrylates) can serve as impact modifiers.^[25]^ Addition of 8 wt % methyl methacrylate‐butadiene‐styrene (MBS) copolymer to TPA‐CHDM/TMCD copolyester gave 892 and 703 J/m notched Izod impact strength when the compositions were 77/27 CHDM/TMCD and 65/35 CHDM/TMCD in which *cis/trans* ratio of TMCD was 60/40.^[36]^

### 7.2 Flow Modifiers

Branching monomers having three or more carboxylic or hydroxy groups like polyfunctional acids, anhydrides, and alcohols can be added at <1 % to increase strength and viscosity of the melt for the purpose of making polymer foams. Some branching agents are trimellelic acid, trimelletic anhydride, pyromelletic dianhydride, trimethylolpropane, glycerol, sorbitol, pentaerythritol, citric acid, tartaric acid, 3‐hydroxyglutaric acid, 1,2,6‐hexanetriol, and trimesic acid.^[23]^ On the other hand, to reduce the melt viscosity, macrocyclic oligomers can be added for purposes of injection and blow molding. Pressure for injection molding was reduced by 20 % when a 2 % poly(butylene terephthalate) oligomer was added to the TPA‐TMCD/CHDM copolyester.^[32]^

### 7.3 Color Additives

Reactive dyes with hydroxyl or carboxylic groups can be copolymerized to give color. Pigments include titanium white, titanium yellow, carbon black, cyanine blue, chrome green, azo red, and cobalt blue.^[45]^

### 7.4 Slip Additives

Slip additives make it easier to process the plastic melt and the removal from the mold on cooling. They can modify the coefficient of friction by migrating to the interface of plastic and mold that provides lubrication. Examples include waxes, fatty acids, fatty esters, siloxanes, silicones, fluorinated polymers at 0.5–2 %.^[25]^

### 7.5 UV Absorbers and Stabilizers

Some UV additives that can reduce photodegradation include benzotriazole, benzophenone, cyanoacrylates, hindered amines.^[45]^ Adding 10 % Cyasorb 1164, a benzoxazinone UV absorber, reduced free‐radical induced cross‐linking of TPA‐CHDM/TMCD (PCTT) film on UV irradiation.^[39]^

## 8 Applications

Traditionally, BPA polycarbonate has been used for making transparent ballistic armor, but TPA/IPA‐TMCD/CHDM copolyester can serve as a new replacement due to its high toughness.^[8]^ Durable household, kitchen, dishwasher containers, and electronic device parts can be made from these copolyesters due to their high optical clarity. TPA‐TMCD/CHDM copolyesters are more durable laminates on glass because of their higher chemical resistance than BPA polycarbonate.^[6]^ Biomedical equipment like dialysis filter housing^[35]^ and blood therapy containers,^[46]^ and light housings, windows, and films are some applications of these copolyesters. Glass fiber, poly(1,4‐phenylene terephthalamide), Kevlar, carbon fiber, and clay can be added to make composites that may further enhance strength properties.^[8]^ Increase in T_g_ of TPA copolyesters due to TMCD incorporation benefits under‐the hood parts in automotive, aircraft interior parts, insulation cladding, electronic parts and biomedical tubings.^[47]^

These copolyesters can be blended with polyamides, polystyrene, acrylonitrile‐butadiene‐styrene, acrylates, methacrylates, polyetherimides, polyphenylene oxides, polyphenylene sulfides, polyester carbonates, polysulfones, polysulfone ethers, and polyetherketones. TPA‐23–35 % TMCD/EG copolyesters when blended with 25 % recycled PET (rPET) in extruder gave 2.5–2.6 GPa flexural modulus, 46–49 J/m notched Izod strength, 56 MPa tensile yield strength, 52–58 MPa tensile strength at break, 4 % yield strain, 272–325 % break strain, 2.3 GPa Young′s modulus and 87–93 °C T_*g*_.^[25]^ <1 wt % phosphorous compounds like tris‐(2,4‐di‐t‐butylphenyl) phosphite (Irgafos 168) can be added to prevent thermal degradation during melt blending of TPA‐CHDM/TMCD copolyester with PET or BPA PC.^[36]^ The blending of TPA‐EG/CHDM (PETG) with TPA‐CHDM/TMCD produces a translucent blend. Haze increased from 3 to 14 % when the PETG content in PCTT/PETG blend was increased from 5 to 20 %. It has been shown recently that the addition of TPA‐CHDM/EG (PCTG) to the PCTT/PETG blend keeps the haze to <1.5 % on increasing PETG content as it acts like a compatibilizer.^[48]^

## 9 Health Safety of TPA‐TMCD/CHDM Copolyesters

Leaching of BPA from polycarbonate and polyarylate rigid plastics is an acute problem as it is an endocrine disruptor. Therefore, rigid plastics that do not use BPA as a monomer are being produced to improve food and environmental safety. In vitro and in vivo assays have been carried out for many chemicals, FDA, EPA, and OECD databases contain data on disruptive endocrine chemicals (EDCs).^[49]^ TPA and DMT had no reported relative binding affinity (RBA) in estrogenic receptor (ER) competitive binding assay. This assay uses uterine cytosol from Sprague‐Dawley rats to see the ability of a foreign chemical to displace 17*β*‐estradiol in the binding of all ER subtypes.^[50]^ Quantitative prediction from molecular docking of *cis* and *trans* TMCD with the ligand‐binding domain of ER*α* receptor found that they were nonestrogenic.^[51]^ However, in one study, commercially produced TPA‐CHDM/TMCD copolyester (Tritan baby bottle) and other polycarbonate replacement plastics were tested through BG1Luc4E2 and MCF‐7 assays. The BG1Luc4E2 is a reporter gene assay, and MCF‐7 is a breast cancer cell proliferation assay that OECD approves for evaluating EDCs.^[49]^ The copolyester was cut into pieces and stressed by microwave, autoclaving, or UV light. Unstressed and stressed plastics were extracted with saline, 10–100 % ethanol, or distilled water. Only 2 of 6 unstressed, 3 of 10 microwave stressed, and 3 of 14 autoclave stressed products showed significant estrogenic activity (EA), but 23 of 25 UVA (315–400 nm) and UVC (100–280 nm) stressed products showed significant EA.^[52]^ The activation of ER‐dependent signaling could have been due to copolyester′s degradation products or additives, like triphenylphosphate (TPP) which has EA.^[53]^ In a study, Tritan baby bottles were incubated with milk simulant (50 % ethanol) at 70 °C for 2 h using standard methods for food contact materials to identify migrants by LC‐MS. Only a slip additive (erucic amide) was detected in the simulant.^[54]^ One study found that survival of B cells of the immune system was better with DMT, TPA, TMCD, and CHDM than BPA.^[55]^ However, a recent study found that DMT, TMCD, and CHDM at 10 μM concentration inhibited androgenic receptor by 42.3 %, 32.27 %, and 9.95 %, respectively, in fluorescence‐based ligand binding assay. There was no inhibition by these compounds on ER*α* binding assay; however, the % inhibitions on ER*β* receptor were 4.34 %, 9.1 %, and 78.28 %, respectively.^[56]^ Overall, it appears that CHDM and TMCD might show lower endocrine activity than BPA, however, further studies and official testing are needed to get concrete conclusions on their effects on health and environment.

## 10 TMCD Alternatives ‐ CBDO Substituents for Future Copolyesters

**Figure 7:**
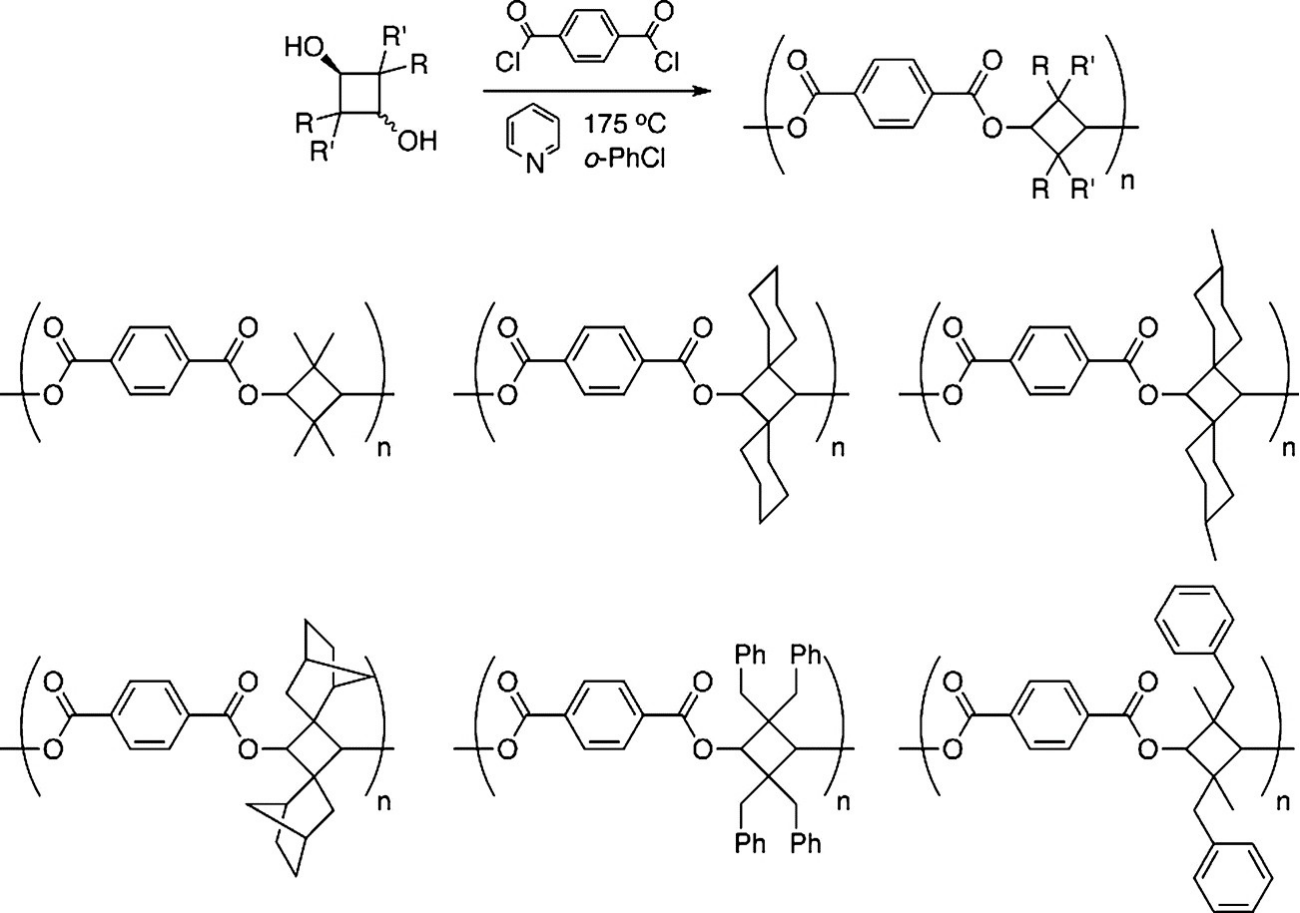
Structurally diverse cyclobutanediols to provide a library of CBDO polymeric materials. Reprinted (adapted) with permission from Burke et al.^[16]^ Copyright 2012 American Chemical Society

The advantage of CBDO as a monomer in polyesters is that it provides alterable side group functionalities. Both linear and cyclic, aliphatic, and phenyl side functionalities have been attached to the CBDO monomer. A range of rigidity and glass transition temperatures of the resultant polyesters can be modified by varying side group functionality like the addition of spirocyclic functionality by the use of 5,5’‐Meldrum acid derivatives. This functionality exponentially increases rigidity and T_g_ (120–230 °C). Moreover, the stereocenters in the spiro side groups add to the number of possible isomers of the substituted‐CBDO which in turn change the glass transition properties of the synthesized polyesters^[16]^ (Figure 7)

The use of cross‐linkers has enabled the alteration of copolyesters based on CBDO monomers. The incorporation of a mild cross‐linking agent has been shown to impact the *T_g_* of the polyester. In one study, the use of 15 mol % phloroglucinol (trifunctional alcohols) in TPA‐60/40 BPA/TMCD copolyester increased *T_g_* from 175 to 193 °C.^[57]^ Alternatively, the use of *cis*‐1,3‐indanediol has also been shown to tune *T_g_* values when used along with TMCD.^[58]^ Isomers add to the possible combinations of chain conformation which affect mechanical properties. The *cis*/*trans* isomer ratio of CBDO can affect the linearity of the polymer chain. Substituents that are also asymmetric further increase the polymer chain′s possible isomers. Specifically, in the case of spiro substituents, combinations of the chain conformation gives a broad range of *T_g_* values while keeping the monomer chemistry constant.^[16]^

**Figure 8:**
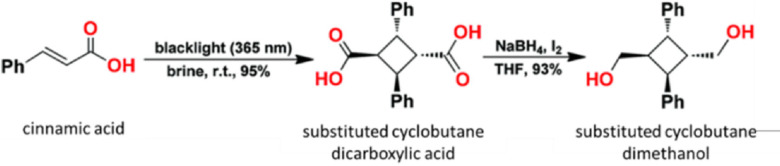
Synthesis of diphenyl cyclobutane dicarboxylic acid (CBDA) and diphenyl cyclobutane dimethanol from *trans*‐cinnamic acid. Taken with permission from Shahni et al.^[59]^

A recent greener approach shows the use of a photoreaction to prepare cyclobutane derivatives. *Trans* diphenyl‐cyclobutane dicarboxylic acid was synthesized from *trans*‐cinnamic acid in a brine medium at 365 nm light (Figure 8). It was then reduced using NaBH_4_/I_2_ in THF to yield *trans* diphenyl‐cyclobutane dimethanol at a 93 % yield. When this cyclobutane dimethanol was esterified with TPA, the copolyester had 23.1 kDa M_w_ and 11 kDa M_n_, and 114 °C *T_g_*.^[59]^ On the other hand, 2,4‐diphenylcyclobutane‐1,3‐dicarboxylic acid (Ph_2_CBDA) could be esterified with linear diols like EG and PDO, but the resulting polyesters had *T_g_* of only 81 and 64 °C, respectively.^[60]^ Nevertheless, these cyclization reactions based on biomass‐derived intermediates like cinnamic acid and furfual^[61]^ can be beneficial for making greener polyesters.

## 11 Summary and Outlook

Polymerization of TMCD in TPA‐based copolyesters improves heat resistance and impact strength. It can be polymerized along with CHDM and linear diols like EG to give material properties that are superior to PET, PETG, PCTG and can serve as a replacement for polycarbonate. There is scope for improvement in the large‐scale synthesis of TMCD as Meldrum′s acid route can be explored instead of vacuum flash pyrolysis. Moreover, many other substituents based on CBDO chemistry can open new doors towards industrially useful copolyesters. Renewable monomers like 2,5‐furandicarboxylic acid (2,5‐FDCA) have been used as TPA substitutes[^62^, ^63^] but the polyethylene furandicarboxylate (PEF) has a T_g_ of 82–89 °C.^[64]^ Therefore, copolymerizing TMCD with FDCA and EG may give a partially renewable copolyester with higher T_g_ than PEF. More toxicological studies are needed to assess the health and safety aspects of TMCD containing TPA copolyesters.

## Author Contributions

SB initiated and contributed to all sections. KB provided content for TMCD synthesis and derivatives. SO and AR helped shape the research and supervise the project.

## Conflict of interest

The authors declare no conflict of interest.

## Biographical Information

*Dr. Samarthya Bhagia is a postdoctoral research associate in the Biosciences Division at ORNL. He received PhD in Chemical and Environmental Engineering from University of California, Riverside in 2016 and B.Tech. in Pharmaceutical Chemistry and Technology from Institute of Chemical Technology, Mumbai in 2011. He is currently investigating 3D printing of green polymer composites along with synthesis and characterization of a variety of plastics. He has deep experience in process engineering of lignocellulosic biomass into renewable fuels, chemicals and materials, characterization of polysaccharides and lignin, and enzyme kinetics*.

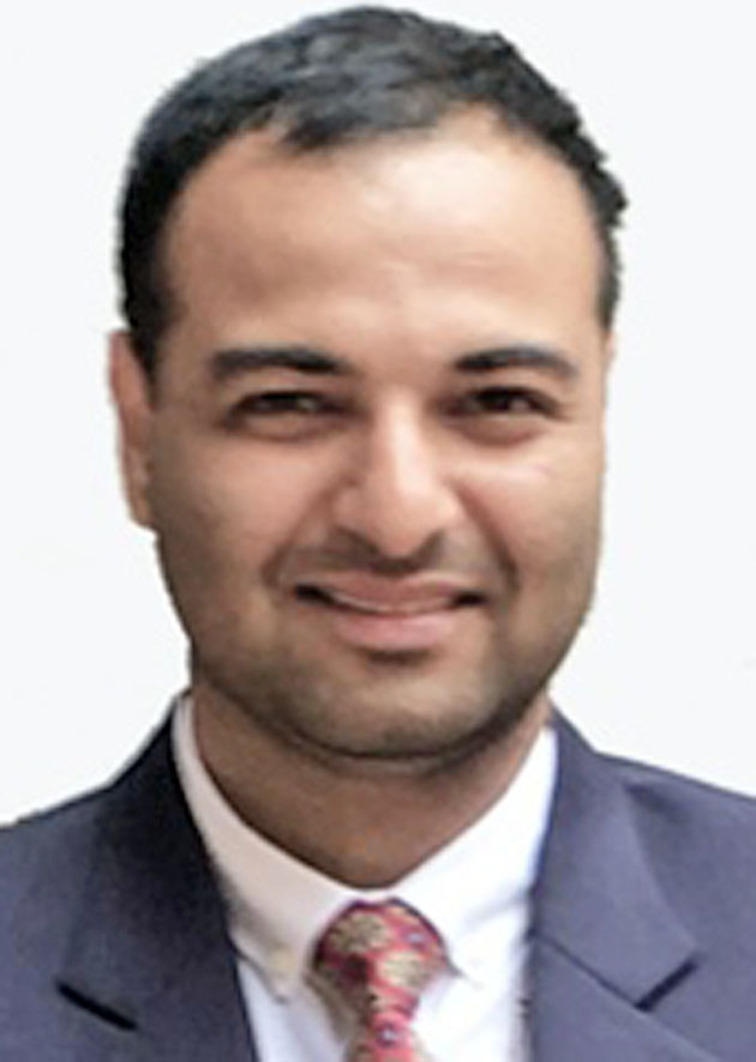

## Biographical Information

*Dr. Kamlesh Bornani received his Ph.D. in Polymer Chemistry at the University of Tennessee Knoxville in 2016 under supervision of Dr. Michael Kilbey. His work focused on characterizing block copolymers in solution, blends and nanocomposites. He then transitioned to work in carbon fiber composites in association with IACMI industry partners. Since 2019 he is working with Dr. Linda Schadler at the University of Vermont. His research interests include understanding the interrelationship between crystallization and nanofillers organization in composites (nano & bio). He is actively pursuing characterization of these composites using novel tools such as AFM and Raman*.

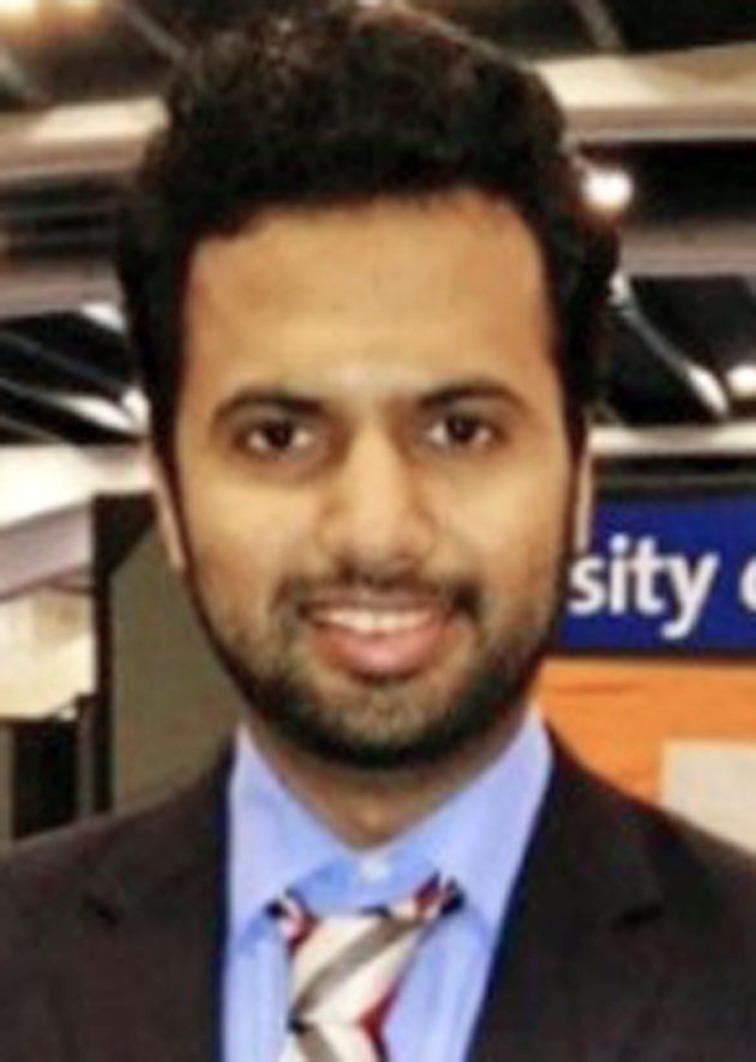

## Biographical Information

*Dr. Soydan Ozcan is a Senior R&D Scientist of Oak Ridge National Laboratory (ORNL). Ozcan is currently the Thrust Lead for Sustainable Manufacturing Technology at the ORNL's Manufacturing Demonstration Facility (MDF). His research addresses the broad and vital manufacturing issue that enables carbon‐neutrality and reduced energy intensity by reducing material waste, promoting complete recyclability and circular economy of materials, advancing the use of bio‐derived renewable resources. Ozcan has received multiple prestigious awards, including R&D 100 – (2020), CAMX Outstanding Sustainability Paper (2018), CAMX Ace (2017), ORNL SEA (2017 and 2020), UT‐Battelle's R&D Award (2012). Ozcan holds 21 patents, has published 9 book chapters, and has been an active speaker sustainable manufacturing and materials‐related topics and research*.

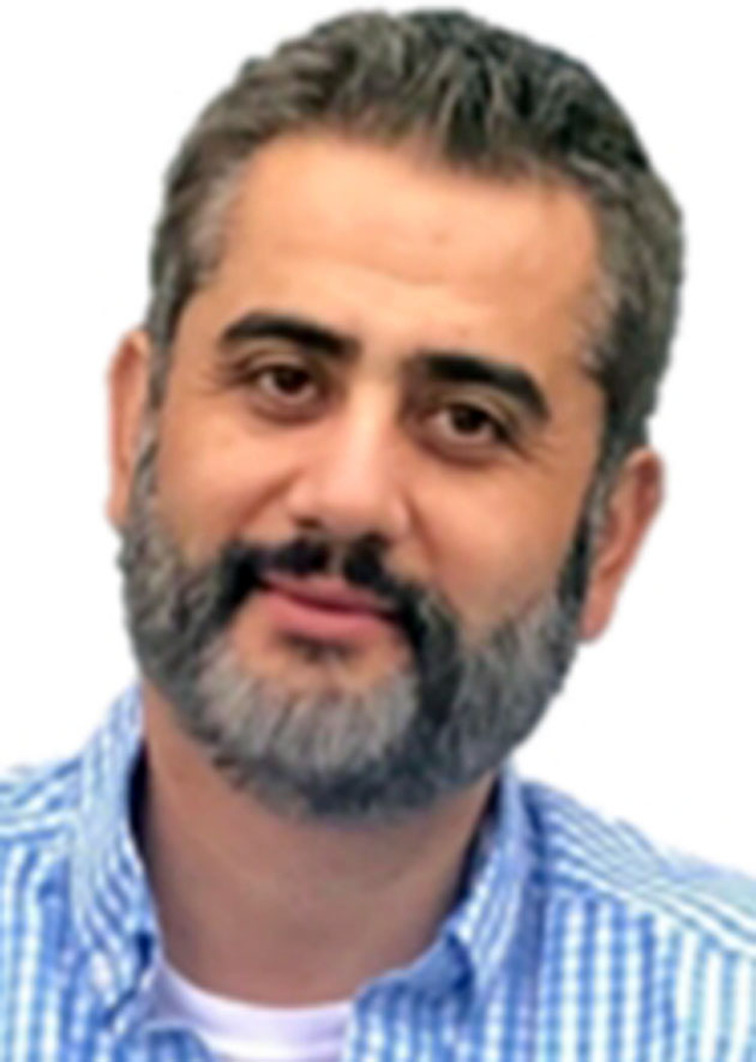

## Biographical Information

*Dr. Arthur Ragauskas is a Governor's Chair in Biorefining at the University of Tennessee. He held the first Fulbright Chair in Alternative Energy and is a Fellow of American Association for the Advancement of Science, the International Academy of Wood Science and TAPPI. His program is directed at understanding and exploiting innovative sustainable bioresources, which targeted to develop new and improved applications for nature's premiere renewable biopolymers for biofuels, biopower, and bio‐based materials and chemicals. More information on his research can be found at**http://cbe.utk.edu/people/art‐j‐ragauskas*.